# CRISPR-Based Programmable Nucleic Acid-Binding Protein Technology Can Specifically Detect Fatal Tropical Disease-Causing Pathogens

**DOI:** 10.1155/2022/5390685

**Published:** 2022-07-18

**Authors:** Md. Rashidur Rahman, Toma Rani Majumder, Md. Aminul Islam Apu, Alok K. Paul, Afrina Afrose, Biplab Kumar Dash

**Affiliations:** ^1^Department of Pharmacy, Jashore University of Science and Technology, Jashore 7408, Bangladesh; ^2^Department of Genetic Engineering and Biotechnology, Jashore University of Science and Technology, Jashore 7408, Bangladesh; ^3^Department of Biotechnology and Genetic Engineering, Islamic University, Kushtia 7003, Bangladesh; ^4^Department of Biotechnology and Genetic Engineering, University of Development Alternative, Dhaka 1207, Bangladesh; ^5^Department of Pharmacy, BRAC University, Dhaka 1212, Bangladesh

## Abstract

Diagnostic approaches capable of ultrasensitive pathogen detection from low-volume clinical samples, running without any sophisticated instrument and laboratory setup, are easily field-deployable, inexpensive, and rapid, and are considered ideal for monitoring disease progression and surveillance. However, standard pathogen detection methods, including culture and microscopic observation, antibody-based serologic tests, and primarily polymerase chain reaction (PCR)-oriented nucleic acid screening techniques, have shortcomings that limit their widespread use in responding to outbreaks and regular diagnosis, especially in remote resource-limited settings (RLSs). Recently, clustered regularly interspaced short palindromic repeats (CRISPR)-based programmable technology has emerged to challenge the unmet criteria of conventional methods. It consists of CRISPR-associated proteins (Cas) capable of targeting virtually any specific RNA or DNA genome based on the guide RNA (gRNA) sequence. Furthermore, the discovery of programmable trans-cleavage Cas proteins like Cas12a and Cas13 that can collaterally damage reporter-containing single-stranded DNA or RNA upon formation of target Cas-gRNA complex has strengthened this technology with enhanced sensitivity. Current advances, including automated multiplexing, ultrasensitive single nucleotide polymorphism (SNP)-based screening, inexpensive paper-based lateral flow readouts, and ease of use in remote global settings, have attracted the scientific community to introduce this technology in nucleic acid-based precise detection of bacterial and viral pathogens at the point of care (POC). This review highlights CRISPR-Cas-based molecular technologies in diagnosing several tropical diseases, namely malaria, zika, chikungunya, human immunodeficiency virus and acquired immunodeficiency syndrome (HIV-AIDS), tuberculosis (TB), and rabies.

## 1. Introduction

A rapid and precise diagnosis is vital for effectively controlling an infectious disease outbreak. Nucleic acid-based pathogen detection has frequently been used in clinical laboratories because of its specificity, sensitivity, and robustness. PCR, which can detect target nucleic acid from a trace amount of clinical samples, has been adopted as the gold standard for nucleic acid-based diagnostics. However, PCR amplification exclusively requires an expensive laboratory setup, expert personnel, careful separate pre-assay preparation time, and post-assay analysis. Advanced isothermal nucleic acid amplification technologies, including helicase-dependent amplification [1], recombinase polymerase amplification (RPA) [2], loop-mediated isothermal amplification (LAMP) [3], rolling circle amplification, and nucleic acid sequence-based amplification (NASBA) [4], overcome the requirement of thermal cyclers, and requires less assay time and cost than the conventional PCR. However, these methods are incompatible, especially for RLSs, because of shortcomings like minimal in-field sensitivity, nonspecific amplification, and inability to detect SNP [5, 6]. CRISPR-Cas systems as the next-generation technologies addressing the shortcomings mentioned above while facilitating POC diagnostic have gained immense interest.

The CRISPR-Cas strategy works as the only adaptive defense mechanism in bacteria and archaea to withstand reoccurring bacteriophage/phage invasion. In general, CRISPR-Cas systems deploy CRISPR RNA (crRNA) or a single-guide RNA (sgRNA)-led Cas endonuclease that sequence-specifically hybridize with the target sites of invader genomes, leading to spotting and cleaving intruder DNA or RNA [7]. Indeed, the flexibility of the CRISPR-Cas system to reprogram its crRNAs for recognizing and editing any nucleic acid sequence has made this tool exquisitely powerful to use in various cells and organisms with immense diagnostic and therapeutic potential [8–10].

Based on the evolutionary relationships, CRISPR-Cas systems can be grouped into two classes (class I comprises multiple effector proteins while class II has a single crRNA-binding protein), six types, and over 30 sub-types [11]. Several in-depth reviews have covered the characteristics of different CRISPR-Cas systems [6, 12, 13]. In the case of type II CRISPR-Cas9 system (Cas 9 from *Streptococcus pyogenes*, *S. thermophilus*, *Staphylococcus aureus*, *Neisseria meningitidis*, and *Campylobacter jejuni*), an RNA duplex formed by a trans-activating crRNA (tracrRNA) bound crRNA fuses with the hairpin-rich region of a 20-nucleotide long sgRNA that is complementary to a protospacer region of the target double-stranded DNA (dsDNA) sequence [7]. Successful recognition and binding of the target sequence by sgRNA brings Cas9 into close proximity to the target and unleashes its nuclease activity, resulting in DNA strand cleavage, forming a blunt-ended double-strand break (DSB) at the target site [7, 14]. Two distinct domains of the Cas9 accomplish this target-specific cleavage; the His-Asn-His (HNH)-like nuclease domain breaks the DNA strand complementary to the target strand while the RuvC-like nuclease domain cleaves another (nontarget) strand of the dsDNA duplex [15]. The DSB is then repaired by the most frequent and efficient error-prone nonhomologous end joining or by the high-fidelity homology-directed repair pathway resulting in particular genome editing at the DSB site using a homologous repair template [7, 14]. Indeed, Cas9 can be engineered to recognize virtually any single-stranded DNA (ssDNA) or RNA (ssRNA) sequence by introducing a protospacer adjacent motif (PAM)-presenting oligonucleotides sequence located 3-4 nucleotides downstream of the protospacer [16]. The recently reported Cas9-based leveraging engineered tracrRNAs and on-target DNAs for parallel RNA detection (LEOPARD) method can simultaneously detect RNAs with single-nucleotide specificity from different viruses in a single run in patient samples [17].

**Figure 1:**
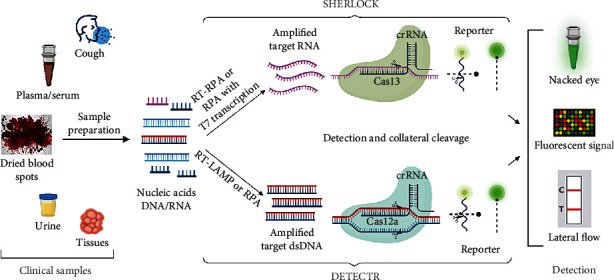
CRISPR-based nucleic acid detection assays employing the Cas trans-cleavage activity (SHERLOCK and DETECTR). Clinical samples after initial preparation for nucleic acid (DNA/RNA) extraction, if necessary, are treated for target amplification by isothermal preamplification. Cas nuclease remains inactive if there are no target nucleic acids in the sample. In SHERLOCK technology, target nucleic acids are isothermally amplified by recombinase polymerase amplification (RPA) or reverse-transcriptase (RT)-RPA with either DNA or RNA as input. Amplified DNA is transcribed to RNA by T7 transcription leading to subsequent target-specific activation of Cas13 from *Leptotrichia* sp. (LwaCas13a). Activated Cas13 forms Cas13-crRNA complex leading to on-target RNA cleavage and nonspecific collateral cleavage of off-target ssRNA reporter molecules as well. In the case of DETECTR technology, the sensitivity of target nucleic acids is amplified by RPA or RT-isothermal amplification using loop-mediated amplification (LAMP) for DNA and RNA, respectively. This amplification leads Cas12a (from *Lachnospiraceae* sp. (LbCas12a) or other organisms) to dsDNA targets by a complementary crRNA, resulting in the collateral damage of short ssDNA reporters. The collateral activity of Cas nucleases, in both cases, turns into detectable signals by cleavage of a quenched fluorophore containing reporter nucleotide probes added to the reaction. Quencher separation from fluorophores leads to a stable and robust fluorescent signal detected by several ways like naked eyes, lateral flow readouts, and a fluorimeter (this image was created with https://BioRender.com).

Recent discoveries of several other CRISPR-Cas systems, including type V (Cas12 from *Francisella novicida*, *Acidaminococcus* sp., *Lachnospiraceae* sp., and *Prevotella* sp.) and type VI (Cas13 from *Leptotrichia buccalis*, *L. shahii*, *Ruminococcus flavefaciens*, *Bergeyella zoohelcum*, *Prevotella buccae*, and *Listeria seeligeri*), outstand CRISPR-based systems' effectiveness towards prompt and accurate detection and genome editing of target organisms. Cas12 has two sub-types: Cas12a and Cas12f. Unlike the Cas9 system, Cas12 lacks HNH domain and thereby depends entirely on its RuvC domain to attain PAM-dependent cleavage of target dsDNA [14, 18, 19]. In addition, Cas12 can PAM-independently recognize and collaterally trigger ssDNA cleavage (referred to as trans-cleavage activity) [14]. Cas12f has a comparatively low size range of 400–700 amino acids (aa) than Cas9 (around 1400 aa) and Cas12a (around 1300 aa) and can target both dsDNA and ssDNA with better discrimination of single nucleotide differences in ssDNA than Cas12a [20]. The Cas13 enzyme family (like Cas13a and Cas13b), with a size range of 900–1300 aa, recognizes target ssRNA and exhibits trans-cleavage activity against ssRNA. Cas13a and Cas13b proteins target the protospacer flanking site, a specific nucleotide next to the 3′ end of the protospacer, for their activities [21, 22].  Figure 1 represents a simplified diagram describing two CRISPR-based nucleic acid detection assays (SHERLOCK and DETECTR) employing the Cas trans-cleavage activity.

Next-generation CRISPR-based molecular diagnostics use combined nucleic acid amplification steps with CRISPR-Cas system (Table 1). Specific high-sensitivity enzymatic reporter unlocking (SHERLOCK), one-hour low-cost multipurpose highly efficient system (HOLMES and HOLMESv2), and DNA endonuclease-targeted CRISPR trans-reporter (DETECTR), for example, have been reported in the ultrasensitive detection of DNA and RNA viruses, bacteria, protozoa, and SNP [24, 26, 27, 31–33]. In addition, SHERLOCKv2 facilitates single-reaction quantitative multiplexing with orthogonal CRISPR enzymes (i.e., multiple Cas13 and Cas12 enzymes) that simultaneously detect different targets at zeptomolar (10^−21^ M) concentrations on a portable paper-based lateral flow readout [34]. Another study by Ackerman and colleagues demonstrated combinatorial arrayed reactions for multiplexed evaluation of nucleic acids (CARMEN), a CRISPR-Cas13-based nucleic acid detection platform that can robustly detect 4500 crRNA-target pairs on a single microwell array [30]. Furthermore, combining HUDSON (heating unextracted diagnostic samples to obliterate nucleases) with SHERLOCK has provided a more straightforward detection of pathogens from body fluids like saliva, serum, and whole blood [9]. HUDSON does not require extra nucleic acid extraction steps; it facilitates heat- and chemical-reduction-based lysis of viral particles and inactivation of local nucleases in a sample for better target nucleic acid-endonuclease binding availability. Accomplishing these advanced and ultrasensitive attributes has made CRISPR-based pathogen detection even more appealing in response to rapid diagnosis and surveillance applications worldwide.

Tropical diseases like malaria, TB, and HIV-AIDS are widespread in rapidly expanding areas worldwide. However, a unique group of tropical diseases, including zika, chikungunya, and rabies, are most prevalent in low-income and impoverished populations [35].  Table 2 summarizes the advantages and disadvantages of non-CRISPR-based methods used in diagnosing tropical diseases discussed later in this article. RLSs, like regions where tropical and neglected tropical diseases are common, demand cheap, portable, sensitive, and rapid detection methods for diagnosing the disease-causing pathogens. With the considerably rapid advancement of CRISPR-based detection technologies, they can be employed to detect diseases as mentioned above, meeting the unmet criteria set by the global health respondents like World Health Organization (WHO) and Centers for Disease Control and Prevention (CDC) [36]. This review focuses on the usefulness of the CRISPR-Cas-based platform as an ideal diagnostic and surveillance technology for extensively monitoring tropical diseases, including malaria, zika, chikungunya, HIV-AIDS, TB, and rabies.

## 2. Use of CRISPR-Based Systems in Diagnosing Tropical Diseases

### 2.1. Malaria

Malaria is one of the acute and sometimes lethal vector-borne tropical diseases threatening nearly half of the population of 91 countries worldwide. Female *Anopheles* mosquitoes infected with protozoan parasites from the genus *Plasmodium* transmit this disease to humans. According to the WHO report, in 2020, the projected worldwide malaria cases were 241 million with 627000 deaths, around 14 million more cases with 69000 more deaths than in 2019. According to WHO, the African region contributed to more than 95% malaria cases and 96% malaria deaths alone, of which 80% were children under five years [37]. Among the six known species of the genus *Plasmodium* that can infect humans, *P. falciparum* and *P. vivax* are the most prevalent, accounting for 99.7% and 75% of infections in Sub-Saharan Africa and the Americas, respectively. Although commonly dispersed in malaria-endemic regions, *P. malariae*, *P. ovale curtisi*, and *P. ovale wallikeri* are often understudied. Nonetheless, they can produce uncomplicated vivax malaria-like illness and sometimes may even become fatal if not appropriately treated [38, 39]. *P. knowlesi*, a Southeast Asian long-tailed macaque parasite, zoonotically infects humans, causing more symptomatic malaria with higher parasite counts but similar morbidity to *P. malariae* infections [40].

Like the most deadly diseases, early and species-specific accurate malaria diagnosis effective in RLSs is critical in controlling its spread and preventing further succession and transmission. A person infected with malaria parasites commonly suffers from high fevers, shaking chills, and flu-like illnesses that usually emerge 10–15 days after being bitten by infected mosquitoes. These symptoms are mostly indistinguishable from other viral hemorrhagic fevers, complicating the primary diagnosis [41, 42]. The current malaria diagnostic methods primarily focus on detecting *P. falciparum* infections and their by-products. Other non-falciparum species are often neglected for their detection or remain indiscriminate due to their “less-severe” type infection pattern, thereby frequently underrated for their prevalence and severity [42].

Although light microscopic analysis of stained blood films is treated as the gold standard for malaria diagnosis, shortcomings like high operator dependency and low sensitivity (the typical limit of detection is 100 parasites/*μ*L), among others, restrict its use occasionally throughout much of Africa nowadays. Conventional *Plasmodium*-specific highly expressed histidine-rich protein 2 (HRP2) antigen-based rapid diagnostic tests (RDTs) were used to diagnose 74% of all malaria in Africa in 2015 [37]. Unfortunately, RDTs are ineffective in detecting *P. knowlesi* and asymptomatic malaria with a low number of parasites (under approximately 200 parasites/*μ*l), may produce false-positive results even after resolution of infection, and false-negative results for very high falciparum parasitaemias and parasites with mutated/deleted hrp2 and hrp3 genes [10, 43].

In October 2020, a group of scientists led by James Collins reported a breakthrough field-level optimized malaria assay capable of species-specifically detecting the four most pathogenic *Plasmodium* species: *P. falciparum*, *P. vivax*, *P. ovale*, and *P. malariae* (even *P. falciparum* with mutated/deleted hrp2 and hrp3 genes). This assay utilizes the combined action of an isothermal reverse-transcriptase RPA (RT-RPA) and SHERLOCK to accurately detect symptomatic and asymptomatic malaria carriers by analyzing whole blood, plasma, serum, and dried blood samples [44]. An engineered CRISPR-Cas12a (also known as Cpf1) enzyme is programmed to become active when gRNAs bind explicitly with the target *Plasmodium* dsDNA sequences. A parallel integration of an RT-RPA step that transcribes target RNA into DNA further strengthens the test sensitivity and accuracy by multiplying target dsDNA motifs present in samples. Once activated, CRISPR-Cas12a indiscriminately cleaves nontarget ssDNA with a high turn-over rate of about 1250 collateral cleavage reactions per second. Generation of fluorophore signals due to this nontarget fluorophore-quencher labeled reporter ssDNA degradation helps denote the existence of *Plasmodium*-specific dsDNA in samples is displayed by a plate reader or a handheld fluorimeter. This assay is simple (a fully lyophilized one-pot SHERLOCK protocol that does not require any nucleic acid extraction steps), ultrasensitive (can detect less than two parasites/*μ*l of blood, surpassing the WHO limit of detection in RLSs), and field-applicable (comprised of an optimized 10 min sample preparation step followed by a 60 min parasite detection reaction). In clinical samples, this assay accomplished 100% analytical sensitivity and specificity.

Another recent study by Cunningham and colleagues demonstrates novel proof-of-concept SHERLOCK assays that can robustly detect all human malaria-causing *Plasmodium* species, except *P. knowlesi*, and species-specifically discriminate *P. vivax* and *P. falciparum* [45]. They developed three independent SHERLOCK assays for detecting different *Plasmodium* species, such as the pan-*Plasmodium* SHERLOCK assay (which can detect all five *Plasmodium* species), *P. falciparum* SHERLOCK assay (which can detect *P. falciparum* only), and *P. vivax* SHERLOCK assay (basically can detect *P. vivax* but shows a low-level cross-reactivity for *P. knowlesi*). Unlike Lee et al., this assay programmed LwCas13a to selectively bind to the conserved 18s rRNA genes of human-infecting *Plasmodium* genomes, followed by genus-specific amplification at 37°C for 180 min with fluorescence measurements. Moreover, they reported developing a novel single nucleotide variation detection SHERLOCK assay prototype representing 73% sensitivity and 100% specificity in detecting *P. falciparum* dihydropteroate synthetase single nucleotide variant A581G. Although the prototype showed false-negative results at lower parasite densities, it outperformed amplicon-based deep sequencing in screening sulfadoxine (a primary antimalarial drug)-resistant malaria in RLSs.

Comparing these two published CRISPR-based malaria detection approaches, the assay described by Lee et al. outperforms that reported by Cunningham and colleagues. The former represents superior analytical sensitivity and specificity, a single-pot lyophilized reaction setup, a simple handheld fluorometer or lateral flow readout, requires reduced reaction time, and is cheaper and better suited for field use [44, 45]. Nonetheless, the latter defines excellent clinical trial performance to well-characterized diverse clinical samples and infected mosquitoes for *P. falciparum*. Both diagnostic modalities suggest future upgradation and clinical validation of these potential SHERLOCK-based assays for future use in POC malaria diagnosis, drug-resistance genotyping, and policy decision-making for efficient malaria management in RLSs.

### 2.2. Zika

ZIKV, a Flaviviridae family member, is transmitted to humans mainly by infected *Aedes aegypti* and A. *albopictus* mosquitoes biting. Human ZIKV infection was first documented in 1953 in Nigeria, and several mild human ZIKV-associated illnesses were reported in broad but confined geographical areas of Asia and Africa until the early 2000s [46, 47]. Starting with the Yap outbreak in the Federated States of Micronesia in 2007, mild and severe ZIKV infection has been reported outside of Asia and Africa, including French Polynesia (in 2013 and 2014), South Pacific islands (in 2014–2016), and Australia, Japan, and Norway (in 2014) [48]. In 2015–2016, the Americas observed an unprecedented acute autochthonous zika outbreak that expanded into 48 countries and American territories with more than 17.15 million confirmed cases. Until now, ZIKV infection has emerged as a potential pandemic threat affecting more than 86 countries worldwide with an estimated high risk of new autochthonous transmission, especially in the Americas [49–51].

ZIKV infections are primarily asymptomatic; only around 25% of infected individuals experience common flu-like symptoms, including mild fever, macular or papular rash, non-purulent conjunctivitis, muscle and joint pain, headache, vomiting, and malaise within seven days of infection. These nonspecific zika complications, often shared with dengue or chikungunya infection in areas of co-endemicity, result in misdiagnosis. Moreover, correlations between ZIKV infection and neurological complications like Guillain-Barré syndrome (GBS) and fetal abnormalities, including microcephaly and pregnancy losses, have been observed during the Pacific and Americas outbreaks [52–56].

The standard ZIKV diagnostic tests include serological approaches like IgM-capture ELISA (CDC-MAC-ELISA), Liaison XL Zika Capture IgM test, and InBios Zika Detect IgM Capture ELISA. Nonetheless, high-sensitivity/low-specificity antibody tests are substandard as the zika IgM often cross-reacts with other flavivirus antigens (like West Nile, dengue, and yellow fever) if present in previously infected patient's samples [57, 58]. An additional plaque reduction neutralization test can be used to verify MAC-ELISA results, but several serious shortcomings make its use impractical in LRSs [59]. Although viral particles and RNA have been spotted in breast milk, nasopharynx, serum, urine, vaginal fluids, and saliva of infected individuals, whether target ZIKV antibodies for ultrasensitive detection present in or ZIKV can be transmitted via these secretions are still undiscovered [60–62]. These phenomena and several common inborne drawbacks limit extensive use of traditional nucleic acid-based approaches like RT-PCR, RealTime ZIKA assay developed by Abbott laboratories [63], and Trioplex and primer and probe in-house test developed by CDC [64] in ZIKV detection.

A study by Collins et al. demonstrated a next-generation robust sequence-specific ZIKV detection scheme that attained clinically relevant sensitivity without any significant affinity for the shared dengue virus (DENV) genome [23]. This portable platform deploys in vitro freeze-dried cell-free expression systems combined with an extremely target RNA sensitive NASBA technology and programmable molecular sensors (i.e., RNA toehold switches) to detect the target sequence even in the low femtomolar range (10^−15^ M) from serum samples. Further adaption of Cas9 endonuclease (called as NASBA-CRISPR cleavage) technology provides selective strand break in the synthesized dsDNA only in the presence of a strain-specific NGG PAM that outperforms the former by enabling SNP detection. This advanced synthetic biological approach can differentiate distinct African and American zika strains and closely related ZIKV and DENV [23, 27, 34]. Further validation using laboratory cultured zika strains (spiked into 7% water diluted human serum) and infected viremic macaque plasma (1 : 10 water dilution) exhibited potential POC diagnostic use in LRSs at 3–5 hours running time at the cost of 2–16 USD per transcript. Another study by Myhrvold and colleagues demonstrated a protocol pairing Cas13-based SHERLOCK with HUDSON that can detect region-specific zika strains and DENV directly from patients' body fluids at concentrations as low as one copy per microliter in less than two hours with minimal equipment [9].

### 2.3. Chikungunya

Chikungunya is a neglected tropical disease primarily transmitted by Chikungunya virus (CHIKV)-infected *A. aegypti* and *A. albopictus* mosquitoes (same mosquitoes transmitting DENV) to humans [65]. In the last 20 years, several CHIKV outbreaks have been reported in more than 100 countries worldwide, including Africa, Asia, Europe, and the Indian and Pacific Oceans (https://www.cdc.gov/chikungunya/geo/index.html). Furthermore, co-infection of CHIKV with DENV [66] and CHIKV with ZIKV, DENV, yellow fever virus, or West Nile virus have been reported [67, 68]. Considering its alarming and unprecedented increase in spread in the last decade, WHO listed CHIKV as one of the blueprint priority pathogens (https://www.who.int/blueprint/en/).

Although first spotted in 1952 from the serum of a febrile patient in Tanzania [69], CHIKV's physiology, pathogenesis, transmission, or diagnosis methods are still ambiguous [70]. CHIKV-infected persons experience clinical symptoms substantially similar to those caused by DENV, ZIKA, and malaria parasite, including fever (ranges from mild fever to encephalitis and hemorrhagic fevers), polyarthralgia, myalgia, widespread skin rash, and conjunctivitis [68, 71]. Chikungunya infection at an early age (immediately after the birth) or older age (>65 years) and especially with comorbidities like diabetes and cardiovascular disorders can sometimes be deadly [72].

Lab culturing patient samples may confirm CHIKV infection in the first three days of illness; the requirement of a biosafety level III conditions limits its use in LRSs [73]. Several serological detection approaches, including CHIK-specific IgM antibody test (in serum samples after 5–7 days of symptom onset) and IgG and IgM capture ELISA (in serum/plasma samples better after 3–5 weeks of symptom onset), are available. However, poor-sensitivity, cross-reactivity, and false-positive tests due to co-infection with the Semliki Forest antigenic complex group, such as Mayaro and *O'nyong-nyong* (and other closely related alphaviruses), have been reported [74]. Moreover, samples collected during the first week of illness require an additional CHIKV RNA (in serum or plasma/EDTA sample) detection RT-PCR test for further confirmation. Real-time RT-PCR [75], quantitative RT-LAMP [76], and a single assay for combined detection of ZIKV, DENV, and CHIKV [77, 78] have been reported, but they are of variable sensitivity. Only a molecular reference reagent has received the US Food and Drug Administration (FDA) approval for CHIKV diagnosis so far [73], not any molecular test.

The gene-editing attribute of CRISPR-Cas9 has recently been employed to perform genome-wide screenings to identify and validate two structurally homologous compounds: CD147 [79] and Mxra8 [80], essential for CHIKV entry into host cells. In addition, another report demonstrated the capability of Cas13b nuclease to identify and degrade CHIKV RNA even in mosquito vectors [81]. However, by far the best of our knowledge, any CRISPR-based CHIKV detection method has been reported.

### 2.4. HIV-AIDS

AIDS is a chronic infectious disease caused by the most advanced stage of HIV infection in humans where the infected person gradually becomes immunodeficient as the HIV impairs regular functions of immune cells. AIDS is characterized by increased risks of infectious and oncological difficulties contributing to common comorbidities, including cardiovascular, renal, and hepatic dysfunction. Since being discovered in the 1980s, this virus has created a global pandemic claiming more than 36 million lives, where most of the deceased were from a confined Sub-Saharan Africa region (https://www.who.int/news-room/fact-sheets/detail/hiv-aids). Depending on the clinical symptom presentations, it usually takes several years for AIDS patients to experience serious complications; thereby, diagnostics capable of detecting early phage infections can maximize life expectancy [82].

Depending on the progression of HIV viremia after initial infection, tests including Western blot, ELISA, and radio-immunoprecipitation can precisely diagnose HIV antibodies in a patient's serum or plasma 3–7 weeks after infection [83]. Moreover, an HIV RNA amplification test, NAAT, can screen for HIV-1 infection (but not HIV-2) as early as after one week of illness. However, fourth-generation tests like confirmation and detection of p24 antigen and combined HIV IgG and IgM antibodies have been recommended by the US CDC and European guidelines for HIV testing as standard for acute HIV-1 and HIV-2 diagnosis within 2–3 weeks of infection. These organizations also recommend using HIV-2-specific nucleic acid-based differential tests in samples that tested negative or intermediate HIV-1 infection by standard tests [84, 85]. Several advanced methods, including HIV-2 RT-PCR Kit [86], 5′-long terminal repeats-targeting HIV-2 PCR assay [87], and the latest automated Cobas HIV-1/2 Qual test [88] have been proposed for efficient HIV-1/2 differentiation and confirmation; however, FDA-approved nucleic acid-based differential methods are still in development [89].

A preprint posted in bioRXiv introduced an all-in-one dual CRISPR-Cas12a (AIOD-CRISPR) assay method to detect HIV-1 DNA and RNA with a sensitivity as low as a few copies (https://doi.org/10.1101/2020.03.19.998724). This truly isothermal and robust one-pot reaction assay can detect 1.2 copies of HIV-1 p24 plasmids in just 40 min of incubation. Prospective CRISPR-based next-generation HIV-1/2 diagnostics are likely about to report overtaking the existing standard tests.

### 2.5. TB

TB is *Mycobacterium tuberculosis* (Mtb)-borne bacterial infection transmitted through the air carrying tiny droplets from the coughs or sneezes of an infected person. Although Mtb primarily causes pulmonary disease affecting the lungs, it can harm tissues like the kidney, spine, and brain (https://www.cdc.gov/tb/default.htm). Among a quarter of the world's population infected with Mtb, only 10% progress to active TB disease, where 40% of the total cases remain unnotified and unrecognized. Mtb can be contained in infected persons in a latency period without exhibiting any clinical TB symptoms even for many years [90]. Moreover, the risk of becoming a progressive TB patient multiplies if a person coinfected with immunocompromising conditions. For example, in Sub-Saharan Africa, about 75% of TB patients are coinfected with HIV-AIDS, making diagnosing and monitoring Mtb infection challenging [91]. Nevertheless, WHO keeps inexpensive tools capable of screening and diagnosing Mtb at the early stage of disease and in LRSs at the top of its proposed TB control strategies.

TB testing methods are currently accessible in a variety of forms. Microscopic examination of the presence of acid-fast bacilli in stained sputum smears and gastric aspirate, for instance, can primarily detect Mtb infection with 50–60% specificity (mainly used in low- and middle-income countries). Traditional tests like Mantoux tuberculin skin test, TB blood test, and interferon-*γ* release assays (IGRAs, in low *tuberculosis* prevalence settings) cannot distinguish between latent or progressive TB and demand additional confirmatory tests like chest radiography and computer-aided imaging. Among the rapid Mtb nucleic acid diagnostic tests, WHO recommends using Xpert MTB/RIF Ultra and TB LAMP. Together with other technologies endorsed by WHO, these tests proved effective in diagnosing HIV-associated and different resistant forms of TB [92–96]. However, their roll-out has barely improved the global TB detection rates since they are sophisticated and expensive with limited effectiveness [90].

A CRISPR-MTB test approach has been reported to detect Mtb in direct clinical samples, including sputum, bronchoalveolar lavage fluid, CSF, pleural fluid, ascites, and pus with improved sensitivity (i.e., with a near single-copy), less sample input, and quicker turnaround time than GeneXpert MTB/RIF and culture assays [97]. Another recent study developed a successful species-specific gRNA-driven Cas12a-based Mtb rpoB sequence detection platform capable of detecting human Mtb and six nontuberculous mycobacterial species (clinical isolates) in three hours without any false-positive results [98]. Lyu et al. emphasized the potential of using the CRISPR platform in low-cost, rapid, and highly responsive diagnosis of pediatric TB as well [99].

Zhang and colleagues introduced an engineered nuclease deficient Cas9 (dCas9)-based in vitro DNA detection system that can reliably detect the human Mtb genome while preserving the functional repertoire of nucleic acids [100]. They demonstrated a pair of dCas9 reporter proteins heterodimerized with the N- and C-terminal cleaved firefly luciferase enzymes guided by single-guide RNAs to complement ∼44 bp upstream and downstream target sequences, respectively. The presence of the target DNA sequence leads the two segments to reside nearby, followed by the catalytic activity of luciferase to create and measure luminescence signals in a lateral readout. This technology is promising in clinical RLSs in multiplex detection of low concentration target genomes even when samples become contaminated with ample nonspecific DNA.

### 2.6. Rabies

Rabies is a neglected tropical disease caused by rabies virus (RABV), a negative-sense single-stranded neurotropic virus belongs to the *Lyssavirus* genus in the *Rhabdoviridae* family. As a zoonotic disease, rabies is endemic in most parts of the world, particularly in resource-limited countries in Africa and Asia, taking around 60000 human lives annually [101, 102]. RABV is naturally transmitted via infected animals' saliva (transmission by tissue plantation has also been reported [103]), and infected dog bites are accountable in more than 99% of reported human cases. Early clinical symptoms of rabies infection are primarily nonspecific, including fever with pain, tingling, and burning sensation in the bitten areas. Although clinically indistinguishable from GBS and cerebral malaria, RABV-specific symptoms only appear when the virus replicates in the central nervous system [104]. Once the virus infects the human brain, it causes acute encephalomyelitis with a nearly 100% mortality rate, as there is no effective treatment [103].

Commonly used tests to detect RABV infection include viral nucleocapsid antigens-specific rapid immunohistochemistry and direct fluorescent antibody tests (FAT), and viral RNA-specific RT-PCR (even RT-PCR-ELISA) [105–107]. FAT is generally valid for *postmortem* cases since viral antigens reach to detectable range only at the final phase of the disease. In the case of intra-vitam human rabies routine diagnosis, conventional and real-time quantitative RT-PCR targeting the highly conserved viral N or L gene has been employed widely. These detection methods have similar sensitivity and specificity for RABV and are valid for detecting mild to severe clinical symptoms bearing host samples [108]. Nonetheless, besides the typical drawbacks, these methods are invalid in the early stages of infection when the viral loads are low, and only a few of these methods have been validated for widescale use in clinical conditions [109, 110].

Recently, an RPA-CRISPR-Cas13a approach demonstrated viral RNA detection from a model rat's cerebrospinal fluid (CSF) that paves the way for early-stage (i.e., three days post-infection) RABV detection [111]. This early rabies detection method has superior sensitivity, detecting as low as a single copy of RABV genomic RNA per microliter CSF. However, a few limitations, including yet to apply with human samples in clinical settings and the requirement of a rigorous primer screening before sample analysis, demand further improvement and validation of this method in using in RLSs.

## 3. Conclusion

The CRISPR-Cas-based detection tools have revolutionized modern medical facilities, especially with their increasingly inexpensive, precise, ultrasensitive, multiplexing, and robust pathogen detection strategies. These tools hold pronounced perspectives for POC applications and routine clinical care in remote LRSs, basically where novel and recurrent tropical outbreaks occur. Applications of cell-free synthetic biological approaches have made these tools even more attractive to use in a low-cost manner allowing for easy storage and distribution in global settings. Moreover, the freeze-drying paper-based platforms overcome mandatory laboratory settings without concern over biosafety. Nonetheless, most of the next-generation CRISPR tools have not yet been validated beyond proof-of-concept applications in real-world clinical conditions and have not been officially approved by the regulatory agencies for extensive field-level uses. Considering their potential, we believe overcoming these limitations is a mere matter of time, and further development of CRISPR-based diagnostics will contribute to challenging global health crises in convincing ways.

## Figures and Tables

**Table 1:** Comparison of CRISPR-based diagnostics with nucleic acid preamplification steps used in detecting tropical diseases.

Method	Enzyme	Amplification	Applications	Assay time (min)	Readout	Target	PAM specificity (based on CRISPR types)	Sensitivity	Cleavage pattern (based on CRISPR types)	References
NASBACC	Cas9	NASBA	Discrimination between African and American Zika strains	120–360	Colorimetry	RNA	3′-NGG (SpCas9), 3′-NNGRRT (SaCas9), 3′-NNNNGATT (NmCas9)	fM	Blunt	[23]
HOLMES	Cas12a	PCR	Discrimination of SNP and virus strains	105	Fluorescence	DNA, RNA	5′-TTTN or 5′-TTN, or 5′-YTN	aM	Staggered	[24, 25]
HOLMESv2	Cas12	LAMP	Discrimination of SNP, detection of RNA viruses and DNA methylation	75–120	Fluorescence	DNA, RNA		aM		[26]
DETECTR	Cas12a	RPA	Detection of HPV16 and HPV18	60–120	Fluorescence	DNA		aM		[14]
SHERLOCK	Cas13	NASBA or RPA	Detection of ZIKV and DENV, and several bacteria and discrimination of SNP and virus strains	180–300	Fluorescence	DNA, RNA	3′-H (LshCas13a), 5′-D, and 3′-NAN or 3′-NNA (BzCas13b)	aM	Near U or A	[27]
SHERLOCKv2	Cas12, Cas13	RPA	Detection of ZIKV and DENV, and several bacteria and discrimination of SNP and virus strains	60–180	Fluorescence or lateral flow	DNA, RNA		zM		[28, 29]
CARMEN	Cas13	PCR or RPA	Detection of 169 viruses, including SARS-CoV-2, HIV, Influenza, and Zika	200	Fluorescence	DNA		aM		[30]

NASBA-nucleic acid sequence-based amplification; RPA-recombinase polymerase amplification; LAMP-loop-mediated isothermal amplification; HPV 16 and 18-human papillomavirus sub-types 16 and 18; SARS-CoV-2-severe acute respiratory syndrome coronavirus 2; fM-femtomolar (10^−15^); aM-attomolar (10^−18^); zM-zeptomolar (10^−21^).

**Table 2:** Advantages and disadvantages of non-CRISPR-based detection techniques used in diagnosing tropical diseases.

Tropical diseases	Diagnostics	Advantages	Disadvantages
Malaria	Light microscopy	The gold standard for Malaria diagnosis	High operator dependency and low sensitivity
	Rapid diagnostic tests	Widespread use in Africa	Chances of false-positive and false-negative results
Zika	Antibody-based serological tests	Readily available	False-positive results because of cross-reactivity with other flavivirus antigens
	NASBA	RNA sensitivity	Inability to detect SNP
Chikungunya	Serological test	Availability	Poor sensitivity, cross-reactions, and false-positive results
	Rt-PCR, RT-LAMP	Real-time detection	Variable sensitivity
HIV-AIDS	Western blot, ELISA, and radio-immunoprecipitation assay	Portable, POC testing	Inaccurate test results because of the window period
	NAAT	Early diagnosis	Capable of detecting only HIV-1
Tuberculosis	Microscopy and traditional tests	Low cost and availability	Low specificity, inability to discriminate latent or progressive TB
Rabies	Rapid immunohistochemistry and direct fluorescent antibody tests, RT-PCR	Valid for detecting mild to severe clinical symptoms	Inability to detect early stages of infection
